# Optimization of Cardiovascular Stent against Restenosis: Factorial Design-Based Statistical Analysis of Polymer Coating Conditions

**DOI:** 10.1371/journal.pone.0043100

**Published:** 2012-08-22

**Authors:** Gayathri Acharya, Chi H. Lee, Yugyung Lee

**Affiliations:** 1 Division of Pharmaceutical Sciences, School of Pharmacy, University of Missouri-Kansas City, Kansas City, Missouri, United States of America; 2 Department of Computer Science and Electrical Engineering, School of Computing and Engineering, University of Missouri-Kansas City, Kansas City, Missouri, United States of America; S.G. Battista Hospital, Italy

## Abstract

The objective of this study was to optimize the physicodynamic conditions of polymeric system as a coating substrate for drug eluting stents against restenosis. As Nitric Oxide (NO) has multifunctional activities, such as regulating blood flow and pressure, and influencing thrombus formation, a continuous and spatiotemporal delivery of NO loaded in the polymer based nanoparticles could be a viable option to reduce and prevent restenosis. To identify the most suitable carrier for S-Nitrosoglutathione (GSNO), a NO prodrug, stents were coated with various polymers, such as poly (lactic-co-glycolic acid) (PLGA), polyethylene glycol (PEG) and polycaprolactone (PCL), using solvent evaporation technique. Full factorial design was used to evaluate the effects of the formulation variables in polymer-based stent coatings on the GSNO release rate and weight loss rate. The least square regression model was used for data analysis in the optimization process. The polymer-coated stents were further assessed with Differential scanning calorimetry (DSC), Fourier transform infrared spectroscopy analysis (FTIR), Scanning electron microscopy (SEM) images and platelet adhesion studies. Stents coated with PCL matrix displayed more sustained and controlled drug release profiles than those coated with PLGA and PEG. Stents coated with PCL matrix showed the least platelet adhesion rate. Subsequently, stents coated with PCL matrix were subjected to the further optimization processes for improvement of surface morphology and enhancement of the drug release duration. The results of this study demonstrated that PCL matrix containing GSNO is a promising system for stent surface coating against restenosis.

## Introduction

Atherosclerosis is a common condition in which plaque built up on the arterial wall results in narrowing and hardening of the blood vessel [1], leading to serious problems like heart attacks, stroke and peripheral vascular diseases. Plaque formation occurs due to accumulation of cholesterol, calcium, lipoprotein and leukocyte on arterial walls [1]. Occlusion of the blood vessel following plaque rupture results in myocardial ischemia and infarction, which can be fatal.

Stent implantation after balloon angioplasty is used to mechanically restore vessel dimensions to ensure a proper blood flow. Stent is a small expandable mesh like structure made of stainless steel, platinum–iridium alloy, tantalum, nitinol, cobalt–chromium alloy, titanium, pure iron or magnesium alloys [2]. In 25–50% of the patients, stent implantation stimulates platelet activation, adhesion, thrombosis and fibromuscular proliferation, producing in-stent restenosis [3], which means re-narrowing of the previously treated blood vessel. Damage to the vessel wall caused by angioplasty procedure and foreign body reactions to the metallic stent seem to trigger immune responses that lead to restenosis.

As pathogenesis of restenosis has been identified to have numerous contributing factors, the regulation of one or two factors is insufficient to control the cascade of the restenosis events. Although numerous treatment options are available for controlling restenosis, a site-specific delivery of immunosuppressant drugs is widely accepted to provide a higher benefit/risk ratio than systemic therapies [4]. Additionally, cardiovascular stents were surface coated with various biodegradable and biocompatible polymers containing immunosuppressant drugs to prevent their escalating incidence of restenosis stemmed from in-stent neointimal hyperplasia.

Majority of cardiovascular drugs are engaged with Nitric Oxide (NO) involved pathways. Since NO is a multifunctional molecule that shows its activity in regulating blood flow, blood pressure and thrombus formation [5], a continuous and spatiotemporal delivery of NO from the surface coated stent at the implanted site could be a viable option to reduce and prevent restenosis [6]–[10]. The development of sustained NO eluting stents poses as a major challenge due to its short physiological half-life. Although numerous studies have been performed to obtain desired therapeutic effects, an optimized NO releasing polymeric platform is yet to be discovered. S-Nitrosoglutathione (GSNO) is a platelet selective NO donor. In patients with severe vascular injury like coronary angioplasty, GSNO treatment has been effective in preventing platelet adhesion and aggregation through NO release mediated by platelet membranes-associated enzymes [11]–[15]. Due to its selective platelet inhibition action as compared to a vasodilatory action, GSNO could be a potential antithrombotic NO donor for the treatment of restenosis [16]. Design of drug eluting stent for a given drug is an attractive, yet tedious process, due to the difficulty in the screening assessment on clinical efficacy of numerous polymeric carrier systems [17]. It is crucial to broaden the horizon on advanced therapeutic research tools to identify suitable delivery systems against restenosis.

Statistical design of experiments (DOE) reduces conventional optimization tasks and helps navigate through critical challenges in the formulation development process. [18], [19]. This technique is widely utilized in industrial setting, as each step offers statistically sound information and provides cost effective solutions [20], [21]. Statistical DOE offers a formidable opportunity to develop new polymeric carriers for NO delivery. Thus, fractional factorial designs are used as a screening tool at the initial stages of biomedical experiments to identify the essential factors that can be investigated more thoroughly in subsequent experiments [22], [23]. The candidate polymers screened using the statistical assessment protocol is further applied for identification of the most suitable coating condition for cardiovascular stent surface.

Poly (lactic-co-glycolic acid) (PLGA), polyethylene glycol (PEG) and polycaprolactone (PCL) are biodegradable, biocompatible, and FDA-approved polymers extensively used for controlled delivery of NO donors [24]–[28]. The polymer system is known to affect the drug release profiles by reducing either drug leaching through the polymeric matrices or the movement of dissolved drug through the polymeric pores/channels [29], [30]. The effects of various formulation properties, such as polymer type, polymer concentration and drug loading amount, on drug release profiles of a model drug and the weight loss rate were evaluated using a two-level full factorial design and residual error analysis. The characterization and optimization process of the polymer system were followed to obtain the most suitable delivery conditions for the therapeutic application.

## Materials and Methods

### Materials

Polymers used for this study were PLGA 50∶50 (DL 2.5A; MW = 25000) (Lakeshore Biomaterials, AL), PEG 4000 (MW = 4000) (Ruger Chemicals, NY) and PCL (MW = 43000–50000) (Polyscience, PA). L-Glutathione (GSH) and all other chemicals were purchased from Sigma, USA.

### Experimental Design

As a preliminary study to screen the polymers, the effects of formulation variables on stent coating efficacy were evaluated based on statistical design of the experimental protocol. Student version of JMP 8.0.2 software (SAS institute, USA) was used to develop the experimental design and to analyze the data [31]. As shown in Table 1, three independent (X1, X2, X3) and two dependent variables (Y2, Y3) were identified. “Screening Design” option in JMP was used to create a design that included the categorical variables that cannot be quantified in the same way as other continuous variables. A two level full factorial design with 12 runs was chosen from the “Design List” and third center points (center values of all factor ranges to check for curvature in the data) was added to calculate the degree of the factor levels, resulting in a total of 15 runs for this study (Table 2). Coded values −1, 0, +1 were used to assign the concentration levels of polymers and drug.

**Table 1:** Independent and dependent variables with their levels in a full factorial design.

Independent Variables		
Polymer (3-Level Categorical); X1	PLGA PCL PEG	
	Low	High
Polymer Concentration (%w/v); X2	5	30
Drug Concentration (%w/w); X3	0.4	0.6
Coded Values	-1	1
**Dependent Variables**		
Weight Loss Percentage; Y1		
Cumulative Percentage Release; Y2		

**Table 2:** The results of full factorial design.

Formulation No.	Pattern^a^	Polymer; X1	Polymer Concentration (%W/V); X2	Drug Concentration (%W/W); X3	Weight Loss (%); Y1	Cumulative Release (%); Y2
1	0−−	PCL	5	0.4	55.59	70.24
2	0−+	PCL	5	0.6	36.24	45.55
3	200	PCL	11.25	0.5	34.32	26.53
4	0+−	PCL	17.5	0.4	30.03	21.38
5	0++	PCL	17.5	0.6	31.24	25.39
6	+−−	PEG	5	0.4	39.99	66.27
7	+−+	PEG	5	0.6	78.89	66.07
8	300	PEG	11.25	0.5	64.83	51.94
9	++−	PEG	17.5	0.4	86.32	71.48
10	+++	PEG	17.5	0.6	86.51	56.02
11	−−−	PLGA	5	0.4	39.81	89.61
12	−−+	PLGA	5	0.6	82.5	71.88
13	100	PLGA	11.25	0.5	39.21	52.73
14	−+−	PLGA	17.5	0.4	32.02	34.88
15	−++	PLGA	17.5	0.6	52.05	30.16

a Pattern: (−) low, (0) Medium, (+) High

### S-Nitrosoglutathione Synthesis

GSNO was freshly prepared for each experiment by modifying previously reported methods [32]. Equimolar concentrations of sodium nitrite and GSH were dissolved in ice cold 0.5 M HCl and stirred for 40 min at 4°C in the dark room to obtain a final concentration of 625 mM. The reaction mixture was added drop-wise to 100 mL of ice-cold acetone with constant stirring. After through stirring for 30 min at 0°C, the mixture formed a pink color precipitate, which was subsequently filtered (through 0.22 µM filter) and washed extensively with pure acetone and diethyl ether. The washed precipitate was vacuum-dried and stored at −20°C until use. GSNO concentration was spectrophotometrically analyzed at an absorbance of 336 nm.

### Preparation of S-Nitrosoglutathione Eluting Stents

Design of experiment (DOE) studies were performed using GSNO eluting stents [33]. First, the required amount of polymer and GSNO were separately dissolved in 2.7 mL of acetone and 0.3 mL of cold DMSO, respectively. The drug solution was then dissolved in the polymer solution and bath-sonicated for 15 min at 4°C. Stents to be coated were cleaned with acetone, ethanol and distilled water in a sequence in bath-sonicator. Cleaned stents were then vacuum-dried for 24 hr. Each stent was coated individually by immersing them completely in test tubes containing GSNO polymer solution for 10 min. The stents were air dried at room temperature followed by freeze-drying at −40°C for 12 hr. For preparing stents with double or triple coat, the second and third coatings were applied on previously air dried single coated stents, followed by freeze drying as the final step similar to that used for single coated stents.

### Preparation of Polymer Films

Characterization of the polymer systems through the conformational studies, such as Fourier transform infrared spectroscopy (FTIR), platelet adhesion and *in vitro* degradation evaluation, GSNO loaded polymeric films were prepared as described in Table 3. Films were casted with the polymeric drug solution (the measured volume of 1 mL) by slowly pouring it into a clean Teflon dish [34]. After the slow solvent evaporation process for 12 hours at room temperature, the films were carefully peeled and freeze dried overnight at −40°C.

**Table 3:** Formulations used for secondary characterization of PCL polymer.

Formulation	Polymer Concentration (%W/V); X2	Drug Concentration (%W/W); X3
F4	17.5	0.4
F5	17.5	0.6
F16	17.5	0.5
F17	17.5	0

### Thermal Analysis

For endothermic thermogram assessment, the pure polymer and polymeric film loaded with GSNO (approximately 1 mg each) were assessed using differential scanning calorimeter (Perkin-Elmer Diamond DSC) [35]. Polymeric film was loaded in teflon dish and vacuum dried, followed by freeze-drying before sealing in aluminum pans. The samples were heated at a rate of 10°C/min from 20°C to 300°C. Instrument was calibrated using known quantity of indium standard at the same scanning rate as used for testing samples. Similarly, the baseline of the thermogram study was recorded using empty aluminum pans. Thermograms were collected for each sample and analyzed (Perkin-Elmer Pyris Software) for peak temperature and area under the thermogram.

### Morphology Assessment of Coated Stent using Scanning Electron Microscopy

Morphologies of bare metal and polymeric coated stents were studied using scanning electron microscopy (SEM) (XL-30, ESEM-FEG, FEI Co., USA) [35]. For this study, bare stents were coated with polymeric film as previously described and stored until they were implanted through balloon expansion. Dried samples were mounted on aluminum pins using double adhesive tapes and gold sputtered under vacuum, and visualized at an accelerated voltage of 80 kV. Samples to be tested for platelet adhesion were also similarly coated and photographed under SEM.

### In Vitro Drug Release Study

The release profiles of GSNO from polymer-coated stents were acquired using total buffer extraction method [35]. Preliminary studies based on DOE (Table 2) were performed using one-time (24 hours) point drug release measurement from stents coated with various combinations of variables including polymer types, polymer concentrations and loaded drug concentrations.

The extended release studies on GSNO eluting stents for a period of 11 days were also performed by incubating samples (prepared as described in Table 3) individually in 1 mL PBS at 37°C and pH 7.4. At predetermined time intervals, the buffer solution was completely removed for quantity analysis of NO and replaced with the same volume of the fresh buffer. The total amount of the drug in each stent was determined by measuring the drug amount left in the stent (i.e., extracting drug from the stent through sonication) at the end of the study period. All the collected samples were filtered with syringe filters (0.22 µM) and analyzed spectrophotometrically at 336 nm. All studies were conducted in triplicates.

### In Vitro Degradation Study on Polymeric Coating Stent

*In vitro* degradation studies were performed on various conditions of polymer coated stents, which were prepared based on descriptions given in Table 2. Stents were immersed in PBS (pH 7.4) and stored at 37°C for three days. After the incubation period, samples were withdrawn, washed, and freeze-dried for 12 hr at −40°C and weighed.

*In vitro* degradation study for an extended time period was performed using polymeric films prepared with various concentrations of the drug as described in Table 3. Dry films were weighed and incubated in PBS (pH 7.4) and stored at 37°C. At predetermined time intervals, films were retrieved and analyzed for the weight loss percentage, which was calculated using the following equation:
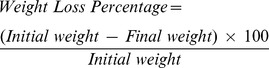



### Assessment of Platelet Adhesion

*In vitro* platelet adhesion study on polymeric films, whose conditions were described in Table 4, was performed using the previously reported method after slight modification [35]-[37]. Whole rabbit blood from New Zealand White rabbits was collected into polypropylene syringe tubes containing acid citrate dextrose A. Collected fresh blood was immediately centrifuged at 1000 rpm for 10 minutes to collect the clear supernatant of platelet rich plasma. Stainless steel disc and samples were incubated in PBS (pH 7, 37°C) for 30 minutes to obtain the surface equilibrium, followed by incubation in plasma for 2 hours at 37°C. Samples were subsequently rinsed with PBS to remove non-adherent platelets and fixed with 2.5% (v/v) glutaraldehyde solution for 2 hours. The samples were again rinsed with PBS and then dehydrated using various dilution ratios of ethanol vs. water solutions (25%, 50%, 75%, 90%, 100%) for 5 minutes each in the order of increasing ethanol concentration. Finally, samples were vacuum-dried in a desiccator and documented as SEM images at 2000x magnification.

**Table 4:** Summary of fit to the model prediction of weight loss percentage and cumulative release percentage.

	Weight Loss (%); Y1	Cumulative Release (%); Y2
R Square	0.923617	0.949617
R Square Adj	0.732661	0.882441
Root Mean Square Error	11.16136	7.125385
Mean of Response	52.63667	52.00867
Observations	15	15

### Fourier Transform Infrared Spectroscopy Analysis

The FTIR spectra for the polymeric films, whose conditions are described in Table 2, were recorded using Nicolet iS10 spectroscope (Thermo Scientific) equipped with an attenuated total reflectance kit. Spectral analysis and superimposition was performed using essential FTIR software with the scanning range from 4000 cm^-1^ to 450 cm^-1^.

### Statistical Analysis

The experimental design and the data analysis were performed using the Student version of the JMP software. Standard least squares were used to fit the models. Data for extended *in vitro* drug release and degradation studies are expressed as mean±standard deviation.

## Results and Discussion

### Statistical Analysis Based Selection of Essential Variables

In this study, a full factorial design was used to screen essential independent parameters. A full factorial DOE is a statistical approach with two or more factors, each with discrete possible levels, and whose experimental units take on all possible combinations of the levels across those factors [38]. This technique allows for studying the effects (impact of changes in factor settings on output) of each factor as well as interactions between factors on the outcome variables [39].

The results obtained from 15 sets of formulations using drug-eluting stents are shown in Table 2. The stepwise regression analysis via JMP software was performed to identify the most influencing factors for each dependent variable. The main effects of one-variable and interaction effects of two variables were taken into consideration with assumption that higher interactions are less significant. The parameters exhibiting the most significant outcomes were selected and the standard least square regression model was applied to fit those parameters.

**Figure 1:**
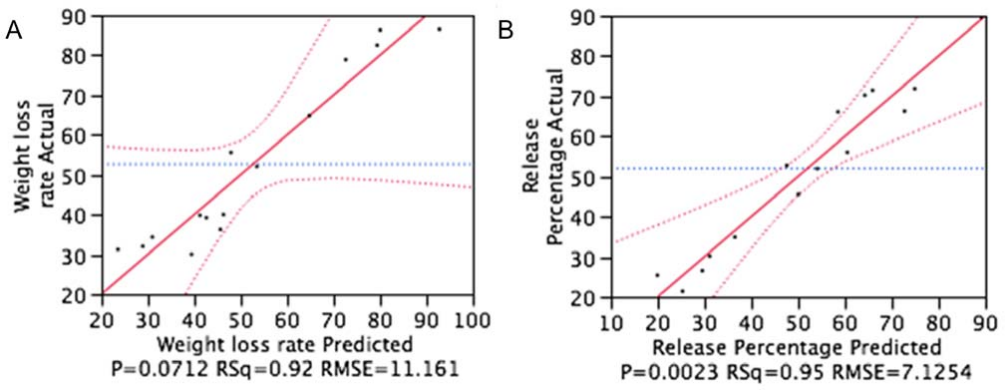
Actuals by predicted plot for weight loss percentage (A) and cumulative release percentage (B).

The actual versus predicted plots of weight loss (%) and cumulative release (%) are shown in Figure 1. A plot of actual versus predicted points is indicative of how effectively the model correlated predicted values with actual values. For a perfect fit model, all the points are supposed to fall on the diagonal line that marks the locus where actual and predicted values are met. The dotted curves represent 95% confidence interval for the data, and the model is said to be significant if this curve crosses the mean values (horizontal) line. In the weight loss (%) plot, the confidence curve does not cross the horizontal line, whereas for the cumulative release (%) plot, the curve crosses the horizontal line, suggesting that the model is well suited with the cumulative release amount. The summaries of fit and analysis of variance were shown in Tables 4 and 5, respectively. The percentage values were used to compare information, since they are sufficient to depict the model fit as a whole.

**Table 5:** Analysis of variance data for the model prediction of weight loss percentage and cumulative release percentage.

Weight Loss (%); Y1				
Source	DF	Sum of Squares	Mean Square	F Ratio
Model	10	6025.4791	602.548	4.8368
Error	4	498.3041	124.576	Prob>F
C. Total	14	6523.7831		0.0712

Cumulative Release (%); Y2				
Source	DF	Sum of Squares	Mean Square	F Ratio
Model	8	5741.6413	717.705	14.1361
Error	6	304.6267	50.771	Prob>F
C. Total	14	6046.268		0.0023*

**Figure 2:**
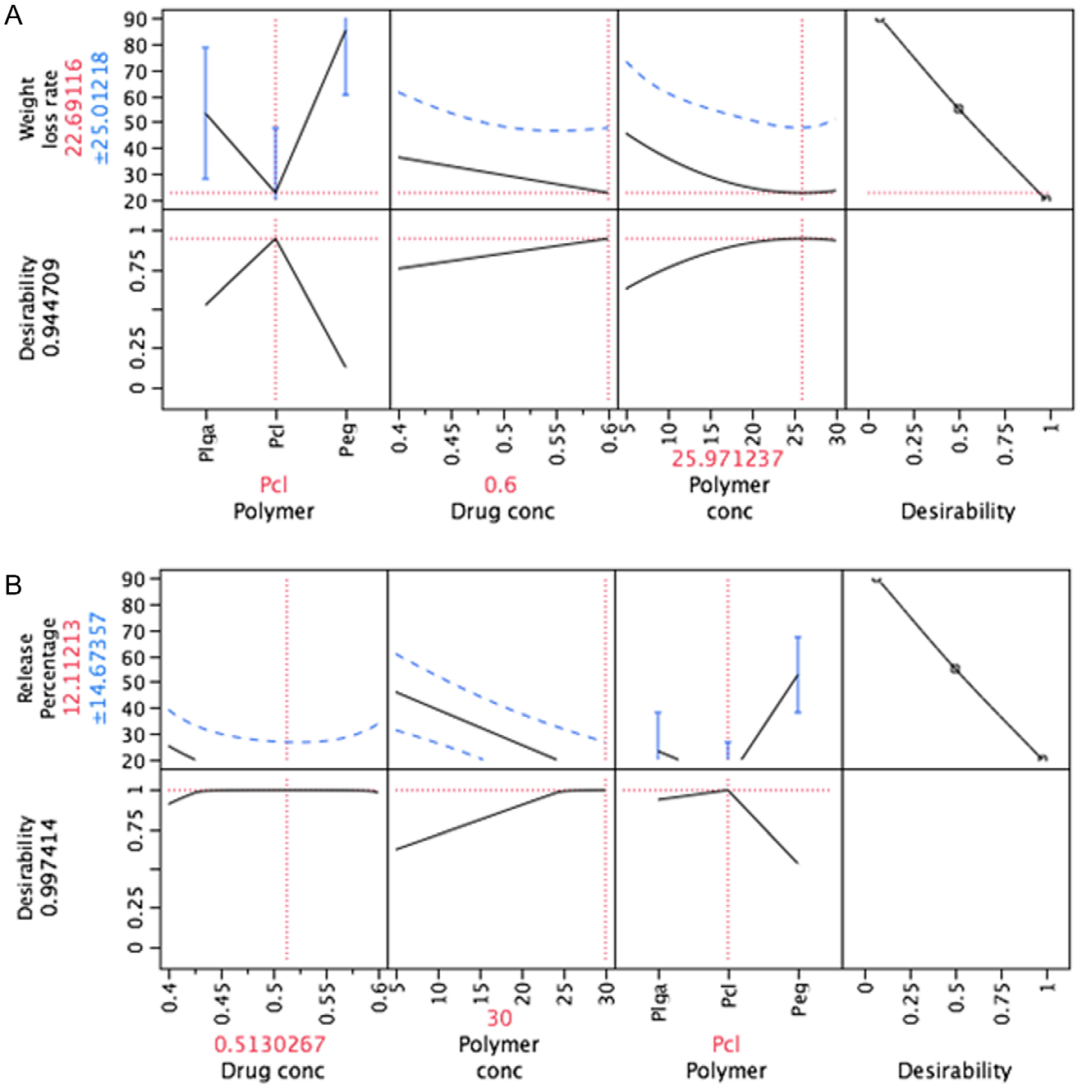
Prediction and desirability plot for weight loss percentage (A) and cumulative percentage release (B).

Desirability plot/profiling is a multi-response optimization method used to simultaneously visualize and optimize the response at varying factor settings. Figure 2A and 2B represent the predicted profiles for the dependent variables based on the model fitting process. The vertical and horizontal dotted lines in these profiles represent X-axis and corresponding Y-axis values, whereas bold lines represent the prediction tracer. These descriptions were utilized in predicting the independent variables and optimizing the required output values. The desirability plot for cumulative release (%) shows that the minimum release level can be achieved by setting independent variables at PCL, 17.5% w/v and 0.5% w/w for X1, X2 and X3 variables, respectively.

The contribution factors of independent variables were assessed based on the slope (i.e., steepness) of the tracer line. In Figure 2B, the tracer line for X3 variable runs parallel to the horizontal dotted lines, implying there were negligible effects of changes in drug concentration on the release profile. For weight loss (%), although the model fit was not significant, the desirability plot for this variable displayed similar settings to those of the cumulative release (%). Based on the experimental design and data analysis, PCL polymer set at the high level of X2 variable (Sample No. F4, F5 in Table 2) was further evaluated for the characterization process of the polymer system for stent surface coating.

### Surface Morphology of S-Nitrosoglutathione Eluting Stents

SEM analysis was performed to study the morphological discrepancies between bare metal stent and GSNO drug eluting stents. SEM images at 300x magnification for a portion of the stent mesh were captured for the assessment of coating thickness and surface topography.

**Figure 3:**
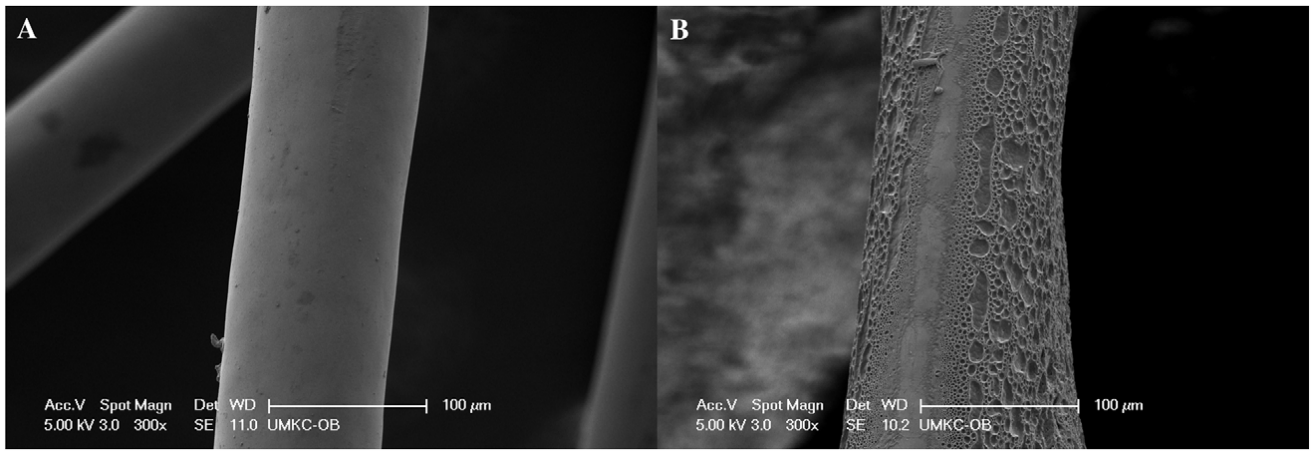
Scanning electron micrographs for uncoated (A) and coated (B) stent surface.

As shown in Figure 3, the differences in SEM images between bare metal stent and drug-eluting stents (DES) were distinctively clear. The polymer-coated surface displayed both irregular pores (voids) and rough coated surface. Physical integrity of the coating surface is fundamental in clinical application of DES. Irregular coating may pose a high risk of friction from the stent during balloon angioplasty procedure, causing a thrombus formation, whereas rough surface can cause damage to the vessel wall. Stent is required to have a smooth surface area to avoid activation of immune responses like platelet aggregation and VSMC proliferation.

As shown in Figure 3, the porous surface of the coating was produced, partially due to the fast solvent evaporation process during the drying process of stents. This irregular, rough and porous surface could be improved by extending the solvent evaporation period. Further studies seem to be needed to find the optimal coating condition and advanced drying methods to overcome porous and irregular surface.

### In Vitro Drug Release Profile

**Figure 4:**
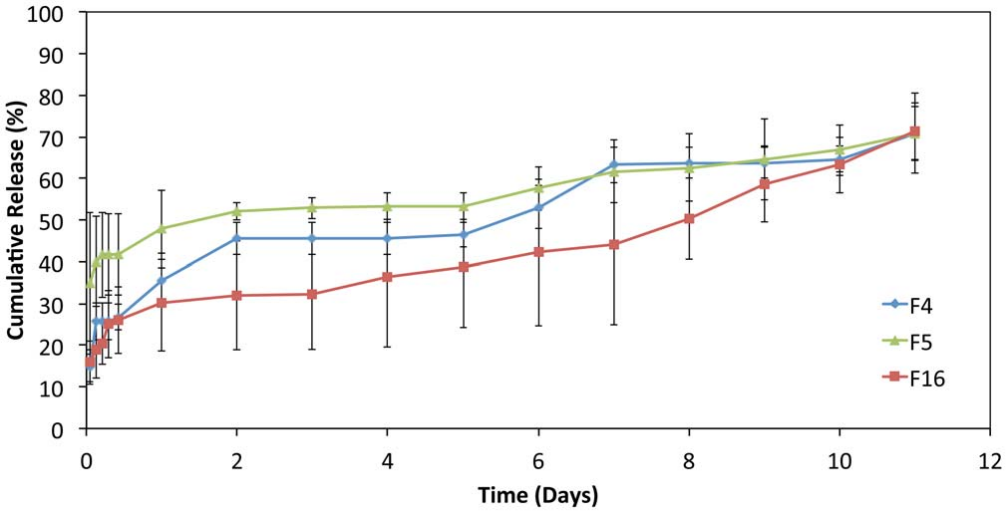
*In vitro* release profiles of GSNO from polymeric-coating stents (pH 7.4, 37°C, n = 3).

The release profiles of GSNO from polymeric coating stents (F4, F5, F16 and F17 stent) are shown in Figure 4. The study was performed over a period of 11 days. The initial burst release amount at day 1 was about 34.5±5.2%, 30.3±11.7% and 47.8±9.2% for F4, F16 and F5, respectively, whereas total release amounts of 70.9±6.3% (F4), 71.2±6.9% (F16) and 70.8±9.7% (F5) of drug, respectively, were obtained over a period of 11 days. The duration of study (11 days) was chosen to depict the region of the profiles containing the initial burst release period followed by the early stage of shaping the steady release patterns.

The results of the preliminary study on the cumulative release amount (%) predicted that the lower drug release rate could be achieved with the stent coated with 17.5% w/v PCL polymer containing 0.5% w/w drug, which was closely compatible with the *in vitro* release amount shown in Figure 4. These results suggested that there was an initial burst release followed by a slow and more controlled release pattern, which are mainly presided by the absorbed drug on surface followed by slow bulk degradation.

**Figure 5:**
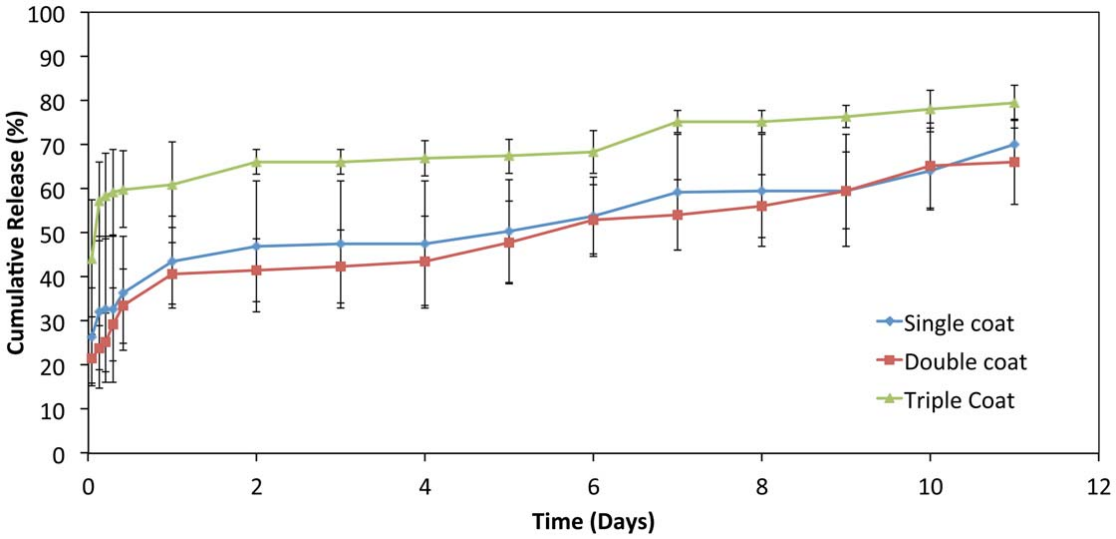
*In vitro* release profiles of GSNO from single, double and triple coated polymeric stents (pH 7.4, 37°C, n = 3).

The effects of multiple coatings on the GSNO release profiles from sample F16 are shown in Figure 5. The profile of the triple-coated stent was marked with an initial burst release of 60.9±9.7%, whereas single and double coated stents showed the released amounts of 43.3±10.5% and 40.7±6.9%, respectively. The burst release of the drug increases proportionally with the number of coatings due to an increase in the drug amount on stent surface. Multiple coatings seem to be ideal for obtaining the sustained release profiles of loaded drugs from polymer coated stents.

The results from the drug release studies suggested that the initial burst release rate increases as drug concentration in the polymer system increases. A slow and linear release profile of GSNO can be achieved by controlling the drug concentration and the number of coatings (i.e., multiple coating) applied to stent surface.

### *In Vitro* Degradation Study

**Figure 6:**
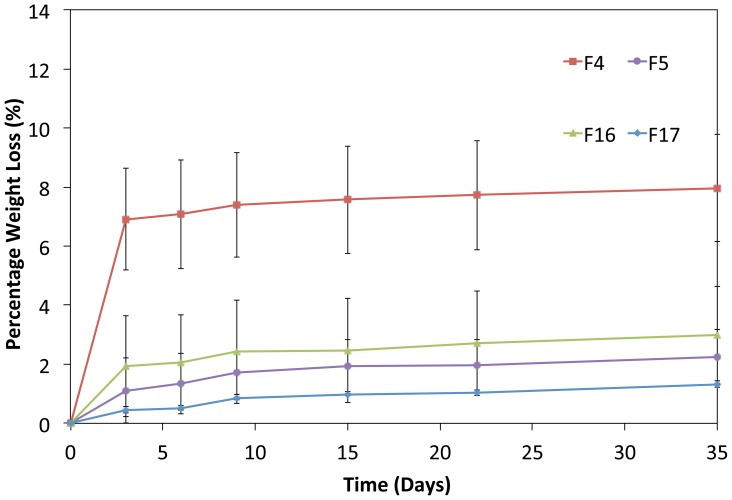
*In vitro* degradation profile from polymeric films of F4, F5 and F16 in PBS (pH 7.4, 37°C, n = 3).

*In vitro* degradation study was performed to evaluate the effects of drug concentration on the polymer degradation rate (Tables 4 and 5). The percentage amount of weight loss for PCL polymeric films loaded with varying concentrations of GSNO was shown in Figure 6. After an initial degradation period of 3 days, the curves for the degradation profiles followed a linear regression with a minimum weight loss for the rest of the study period.

The outcomes of the desirability plot (Figure 2B) for variable Y2 indicated that 0.54% w/w drug concentration was suitable for minimizing weight loss. The results shown in Figure 6 also concurred with this observation. The initial and final weight loss amounts of F5 were about 1% and 2.3%, respectively, whereas those for F16 were 2% and 3%, respectively. Sample F4 had a higher degradation rate than samples of F16, F5 and F17 films. Although the degradation rate of F5 film was lower than those of F4 and F16, the differences were not statistically significantly.

The slow degradation of PCL polymers, both *in vivo* and *in vitro* occurs through the surface hydrolysis process of ester bonds. The weight loss observed in this study seemed to be due to drug diffusion through the films rather than polymer degradation.

### Thermal Analysis of Polymeric Films

**Figure 7:**
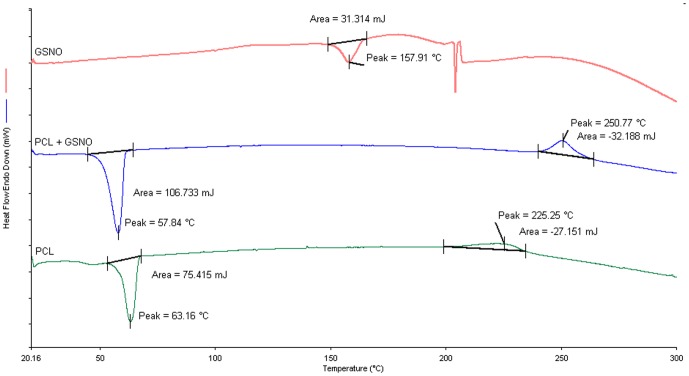
DSC thermograms for PCL films, GSNO and GSNO loaded PCL films.

DSC analysis was performed to evaluate the thermal properties of the samples including pure PCL, GSNO and GSNO loaded PCL film. As shown in Figure 7, an endothermic peak for pure PCL polymer was observed at 63.16°C, implying that the melting point of PCL polymer is within a range of 58 to 63°C. For GSNO loaded film, the melting peak was shifted to lower temperature (57.84°C), which is indicative of the presence of GSNO drug.

The melting temperature for GSNO was monitored at 157.91°C. GSNO drug endothermic peak was not observed in the GSNO loaded film, which may be due to the conversion of the crystalline state of GSNO to amorphous/dispersive state. The results of the DSC study suggested that the addition of GSNO does not affect the thermal properties of the PCL polymer.

### Fourier transform infrared spectroscopy

**Figure 8:**
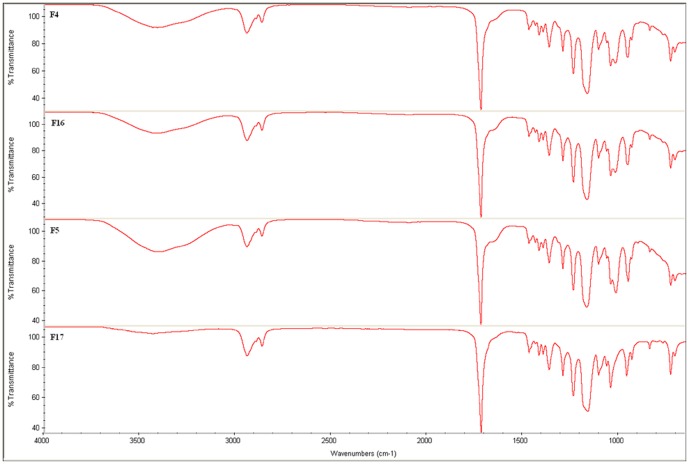
FTIR spectrum of F17, F4, F5 and F16 polymeric films.

The stent surface coated with GSNO loaded polymer was characterized using Infrared Spectroscopy. As shown in Figure 8, the peaks corresponding to the polymer and the drug are distinctively observed from the films of F4, F5, and F16. An increase in intermolecular hydrogen bonding of -OH stretching is observed at 3700–3500 cm^−1^ wavelength in the mixture, suggesting that the added GSNO formed intermolecular hydrogen bonding with the polymer (characterized by –O-H broad stretch). On the other hand, no peak representing intermolecular hydrogen bonding is observed in the polymer PCL alone (F17).

An increase of drug loading in the polymer caused an increase in percentage amount of transmittance of –OH stretch, suggesting there was a direct relationship between drug loading and intermolecular hydrogen bonding.

#### F17

υ_as_ CH_3_ 2890 cm^−1^, C-H stretch 2967, -C = O stretch 1717 cm^−1^, δs CH_2_ C-H bend 1464 cm^−1^, δ_s_ CH_3_ C-H bend 1350, O-C( = O)-C of C-O stretch and bend 1225 cm^−1^, C-O-C stretch 1125 cm^−1^, inplane C-H bend 1050 cm^−1^, out of plane C-H bend 730 cm^−1^.

#### F4, F5 and F16

Stretch –OH intermolecular hydrogen bonding 3700–3010 cm^−1^, υ_as_ CH_3_ 2890 cm^−1^, C-H stretch 2967, -C = O stretch 1717 cm^−1^, δs CH_2_ C-H bend 1464 cm^−1^, δ_s_ CH_3_ C-H bend 1350, O-C( = O)-C of C-O stretch and bend 1225 cm^−1^, C-O-C stretch 1125 cm^−1^, inplane C-H bend 1050 cm^−1^, C-N stretch 1068 cm^−1^, -NH stretch 1020, broad absorption of secondary amines (N-H wagging) from 910–650 cm^−1^, out of plane C-H bend 730 cm^−1^.

### Platelet Adhesion Study

Platelet adhesion study is integral in the assessment of the biocompatibility of implantable materials. Most versatile materials that can be used in medical applications or implants may cause platelet adhesion and activation in contact with blood. For this study, the samples were incubated with rabbit blood plasma. The average platelet size in most rabbit blood is about 1 to 3 µm in diameter.

**Figure 9:**
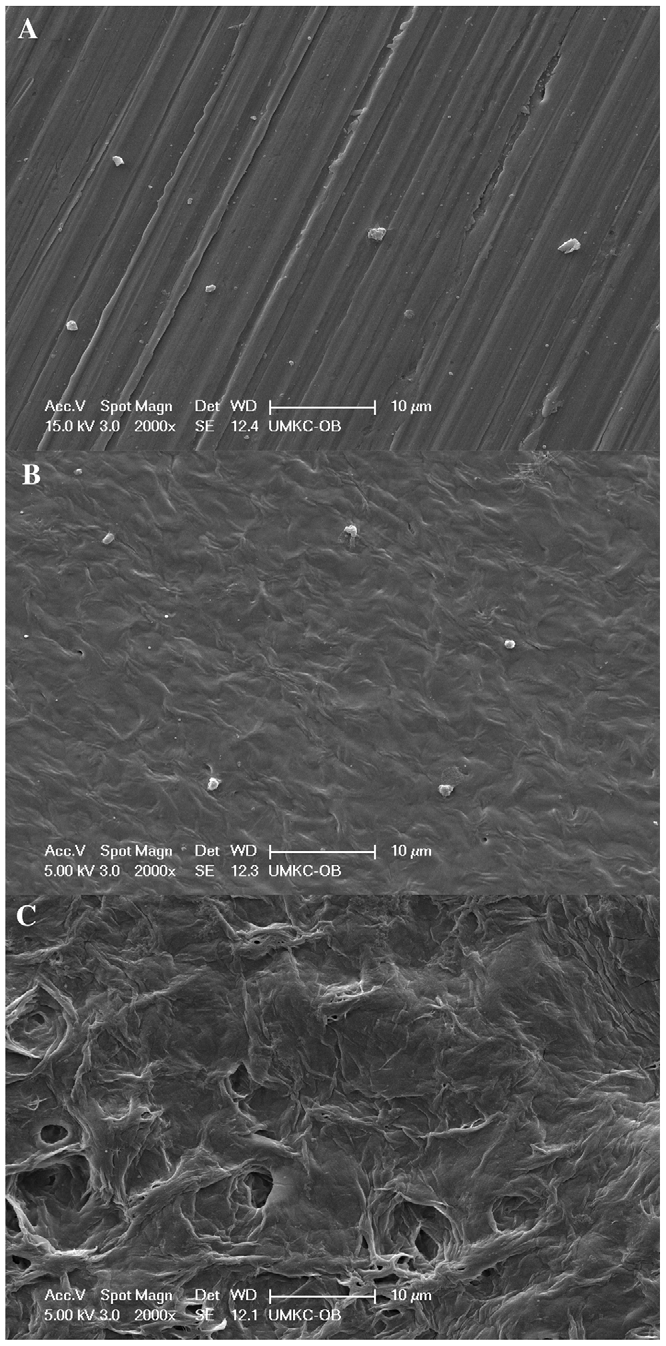
SEM images for platelet adhesion on stainless steel (A), polymer only (B) and drug loaded polymer surfaces (C) (2000X).

As shown in Figure 9, stainless steel plate and F0 film displayed apparent platelet adhesion, whereas the rest of drug eluting films displayed no platelet adhesion. Moreover, there was no difference between films loaded with a low or high GSNO dose (Data not shown), indicating that there was no dose-dependent activity in platelet adhesion within a tested dose range. SEM images for GSNO loaded films showed less porous surface similar to those of the coated stents.

**Figure 10:**
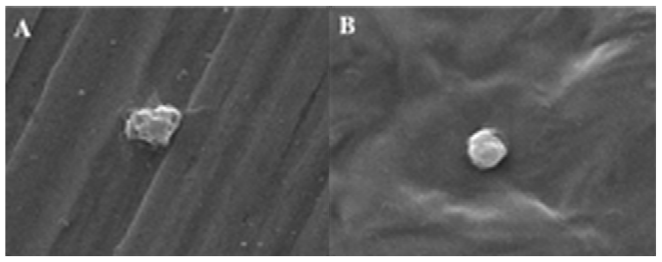
Morphology of platelet adhered stainless steel (A) and polymer film (B).

As described previously, since porous surface poses a higher risk of platelet adhesion and aggregation, drug eluting films with a fewer pores displayed no platelet adhesion. Platelets adhered to stainless steel plate displayed characteristic pseudopodia as shown in Figure 10A, whereas platelets adhered to F0 remained with a round shape (Figure 10B), suggesting that PCL polymer has distinctively improved blood compatibility as compared to bare metal.

The results of the platelet adhesion study show that GSNO eluting films not only prevent platelet adhesion but also activate it, whereas blank films don't. Platelet activation plays a vital role in the cascade of aggregation events that lead to restenosis. Prodrugs of NO, like GSNO, are known to act as an anti-proliferative and an anti-inflammatory agent. Based on this study, it can be concluded that GSNO could serve as an inhibitor of platelet adhesion. This study indicates that there is enormous therapeutic potential of NO-eluting stents against restenosis in its clinical application.

## Conclusion

Statistical experimental design is a rapidly growing field, with promising applications ranging from nanotechnology to aerospace engineering. Although some of the techniques have been applied in pharmaceutical formulations, our group is the first one to combine this approach with polymeric stent coating optimization. The objective of this study was to optimize the physicodynamic conditions of polymeric system as a coating substrate for drug eluting stents against restenosis. Various GSNO-eluting polymeric formulations were prepared using the solvent evaporation method and subsequently assessed via a full factorial design. PCL polymer, high polymer concentration (17.5% w/v; +1) and medium drug concentration (0.5% w/w; 0) of formulation F16, was successfully selected for a prolonged delivery of GSNO. *In vitro* degradation and release studies further supported this selection. The platelet adhesion study confirmed that the GSNO-eluting stent significantly reduced the platelet adhesion and activation. The results of this study demonstrated that GSNO loaded PCL polymer is a promising system for stent coating against restenosis. Further optimization processes are needed to verify the necessary parameters for improvement of coating method, surface morphology and enhancement of the drug release duration of the PCL coated stents.
